# Historiographical approaches to biogeography: a critical review

**DOI:** 10.1007/s40656-023-00580-9

**Published:** 2023-06-22

**Authors:** Alfredo Bueno-Hernández, Ana Barahona, Juan J. Morrone, David Espinosa, Fabiola Juárez-Barrera

**Affiliations:** 1 Facultad de Estudios Superiores Zaragoza, Universidad Nacional Autónoma de México (UNAM), Mexico City, Unidad de Biología Comparada Mexico; 2 Facultad de Ciencias, Universidad Nacional Autónoma de México, Departamento de Biología Evolutiva, Mexico City, Mexico

**Keywords:** Historiography, Biogeography, Biogeographic patterns, Geographical distribution

## Abstract

We performed a critical review of the historiographical studies on biogeography. We began with the pioneering works of Augustin and Alphonse de Candolle. Then, we analyzed the historical accounts of biogeography developed by (1) Martin Fichman and his history on the extensionism-permanentism debate; (2) Gareth Nelson and his critique of the Neo-Darwinian historiography of biogeography; (3) Ernst Mayr, with his dispersalist viewpoint; (4) Alan Richardson, who wrote a microhistory on the biogeographic model constructed by Darwin; (5) Michael Paul Kinch and the ideas discussed in the 19th century about the geographical distribution of living beings; (6) Janet Browne, who highlighted the importance of the pre-Darwinian naturalists; (7) Peter Bowler, who focused mainly on the influence of paleontology on biogeography; (8) James Larson, who looked into the practices of the naturalists of Northern Europe in the late 18th century; and (9) Malte Ebach, who like Larson, was more interested in analysing the practices rather than the ideas of naturalists who studied the geographical distribution of organisms. Finally, these works are compared with each other. There has not been a dominant paradigm in the construction of historical narratives of biogeography; however, they provide a useful context for understanding problems of biogeography that continue to be debated to this day.

## Introduction

Scientific disciplines have emerged mostly throughout the 19th century and have been considered as basic units driving the internal differentiation of sciences (Stichweh, 2006). The definition of discipline has changed over time. In the Middle Ages, discipline definition referred to looking for an order of knowledge for teaching in schools and universities. In the Renaissance appeared encyclopaedias, where disciplines were understood as divisions of it. Finally, in the 19th century, scientific disciplines acquired the connotation of communication systems, conformed to central components of scientific specialization and having a leading role within the structure of science, standardization of scientific publications, and the endless looking for knowledge. In this way, a scientific discipline works like a dominion of teaching and learning in high education and like a central role in both professional and occupational. The interaction among different disciplines represents an essential component of modern science (Stichweh, 2006).

Some studies on the institutionalization process of science departed from the premise of considering it as a cultural production, assuming that scientific disciplines arise from the interplay among components of a community of knowledge, like universities, industries, and the construction of instruments. In this sense, the analysis of experiments, technology, and the intricate relation between institutional, economic, social, and even cultural aspects acquire great relevance (Lenoir, 1997). This point of view contrasts with the positivist conception of science, which gives more importance to the study of the history of scientific theories.

In a more particular sense, the history of a given academic discipline can be understood as the assembly of narratives that describe the transformation and evolution of human cognitive action, which further details the historicity behind the theories it seeks to understand and the criteria of scientific rationality. The history of science has attempted to widen its horizons, and its interest is no longer only centered in the intellectual history of science. Beyond the intrinsic interest that historical studies have in scientific rationality, the historical study of scientific practices and the social construction of knowledge has acquired great momentum, including within itself social, cultural, and political components, thus becoming the study of science history more diverse and specialized. Christie (1990), with a historiographical approach, understood it as the study of the different ways in which the past of science has been written. In the past decades, however, this definition has passed away, and the conception that historiography is no longer only the traditional history of ideas but currently recognizes the importance and impact of the social, technological, and academic environment for scientific practice. The history of particular academic disciplines has had a leading role in legitimizing scientific discipline and the change and adaptation of concepts and methods faced by the historiography of scientific disciplines (Capel, 1989).

Biogeography is a relatively recent discipline, so it should not be strange that there are scarce historiographical studies about it; however, some historiographic narratives have been assembled during the last decades. Herein, we aim to undertake a comparative analysis of a set of histories of biogeography, recognizing the contingent and contextual nature of histories that have been published. This character is not exclusive but shared with general historiography and science. We analyze the main histories of biogeography, developed by both science historians and biogeographers. The authors chosen for this review include Fichman (1977), Nelson (1978), Kinch (1980), Richardson (1981), Mayr (1982), Browne (1983), Larson (1986), Bowler (1996), and Ebach (2015). This is not intended to be an exhaustive revision; however, it compiles noteworthy historical works on biogeography, which display two clearly recognizable stances: one which conceives biogeography as a discipline relevant for the formulation of Darwin´s evolutionary theory, and another that portrays biogeography as an independent self-reliant science.

### Preliminary reflections on the history of biogeography

It seems that historical revisions have been more relevant in time periods when science was breaking the established standards. History provides a frame of reference to situate new ideas and scientific practices within a theoretical context and a social environment. Augustin Pyrame de Candolle (1778–1841) was one of the most active pioneers of 19th century biogeography. His *Essai* is frequently considered the starting point of modern biogeography, at least in the sense of being a critical revision of the ways to recognize the spatial patterns of life from a secular perspective. In his essay, de Candolle takes Linnaean taxonomy as his starting point, as an axis to standardize the knowledge of the flora over the globe.

De Candolle (1820) borrows Linnaeus’ concepts of station and habitation as fundamental elements to analyze the geographic patterns of plants. Unlike the main trend, which portrayed these sections as merely subsidiary to the main purpose of bringing God’s catalogue to completion and discovering the connections amongst its creatures, de Candolle took a special interest in gathering and arranging the huge amount of information contained within them. He noted that only by obtaining in depth information on the physical conditions (station) and on the geographical location where the plants are recollected (habitation), it is possible to recognize accurately the different patterns of the geography of plants. His approach represents an initial detachment of biogeography from taxonomy. He later makes reference to the most important inputs of the main authors who had contributed to the knowledge of worldwide flora.

De Candolle highlights in particular the contributions of the Prussian naturalist Alexander von Humboldt (1769–1859) on methodological aspects, while also putting the new emerging hypotheses about the nexus between the characteristic floras of different botanic regions with fossil floras into perspective:

“Could it be said, according to other naturalists, that the primitive soils must have been the first to be covered with vegetation, having had to precede animal development and, thus, the formation of secondary soils?

In this idea, the primitive parts of the world should have been the centres of the regions; however, asides from the difficulty of recognizing the traces of this dispersion, it is highly doubtful that the plant species that we know today are the same that must have existed before the secondary ones, and from which we find a trail or scraps in these lands.

This intriguing study, launched not long ago, with some degree of precision at least, by Mr. Stenberg, and which Mr. Adolphe Brongniart, as young as the former, seems intended to improve; this study, I say, seems to indicate that our plant species are different to the antediluvian ones, and thus, a new vegetation has developed from the formation of these secondary grounds.

What would happen if we went from these purely geological considerations to those that depend on the bases, and even, to the metaphysics of natural history? All the theory of botanical geography is based on the idea of one single origin of the organized beings and the permanence of species. I will not engage in a discussion on both of these issues here, both possibly unsolvable; but I cannot leave without pointing out their connection with the study of plants distribution” [translated from de Candolle 1820, pp. 58–59].

It is evident that de Candolle breaks away from a ahistorical conception of nature, conceiving biogeographical patterns as the outcome of historical processes.

Alphonse de Candolle (1806–1893), son of Augustin, performed the best revision of the geographical distribution of plants, with a surprisingly externalist approach that was ahead of its time. In three of his core works, Alphonse de Candolle makes a historical revision of the geography of plants, Darwin’s theory, and the development of scientific societies in the late 19th century (de Candolle, 1855). Alphonse de Candolle finds in the works of his father Augustin de Candolle (1820), Humboldt (1805) and Schouw (1823), the most relevant foundations of the modern geography of plants. He finds in Linnaeus’ work the first formal references regarding the origin of plant distribution and he arranges the hypotheses on the nature of the regions and their shared genera as evidence of the historical connection between them. He revisits the debates between a single origin of the flora (i.e., Linnaeus, 1744) against the multiple origins of different floras over the main mountain ranges of the world (i.e., Willdenow & Gmelin, in Browne, 1983; Larson, 1986). Furthermore, he makes a distinction between supernatural hypotheses (based on *Genesis*) and natural ones (based on the evidence and past and present physical conditions). Since then, Alphonse de Candolle pointed out some of the ongoing debates being tackled by contemporary authors (i.e., Kinch, 1980; Larson, 1986).

One of the most interesting points in his work is the criticism of the attempts to perform a historical reconstruction of evolutionary ideas, centered almost exclusively on Darwin (1859), without recognizing other important authors, as well as his defense of Antoine Nicolas Duchesne (1747–1827), whose contribution exceeds the merely idealistic morphology of Goethe (1749–1832) and wraps itself into a clearly genealogical conception of species relationships:

“Several scholars, such as Buchner, Haeckel, Seidlitz, have talked about the precursors of Charles Darwin regarding transformation theory, but none of them mentioned Duchesne. There are several other omissions within the thirty-six names listed in page 57 of Seidlitz, Die Darwinische Theorie, 1875, especially regarding metamorphosis in plants. The first author he points out is Goethe, in 1790, whereas Duchesne precedes him by twenty-six years. Goethe, as Geoffroy Saint-Hilaire and de Candolle would later do, talked about metamorphosis or different states surrounding certain types of mediums; Duchesne addressed the true successive ancestries between generations” (translated from de Candolle 1882, p. 36).

De Candolle (1873) analyzed the development of science during the 18th and 19th centuries. The plural approach with which he addresses the subject stands out for its originality, as it incorporates an array of aspects such as cultural and family heritage, education systems and public policies supporting the scientific community, among others. His sharp judgement even allowed him to foresee the predominance of the English language as the main means of scientific communication, after realizing that the Royal Society displayed an openness that marked a strong contrast with the rigid standards of its peers in Berlin and Paris. In short, Alphonse de Candolle developed a historical reconstruction of biogeography with an externalist approach, in which he assigned a fundamental role to the academic community in the reception and approval of new models, so long as these were conceived with facts and more meticulous methods.

“There are times when the old ideas start to become worn out, or when certain methods are no longer sufficient, when scientists working a certain field start feeling uneasy and long for something new….

I refer to the memories of those who worked and brought new ideas forward back in 1859, the same year the work *On the Origin of Species* was published. The science department was at risk. New facts were bringing it down from several angles. Therefore, the species descriptors were no longer sure what to think of the species, which seemed to be meant to be considered as defined, almost unchanging, groups for a long time, produced thousands of years ago, for reasons mankind would not be able to comprehend. According to several distinguished scientists, natural history has the sole objective of studying these groups, their organs as we see them, their life form and their similarities, defining their heritage from a superior order. What had preceded them, what could come afterwards was unavoidably hypothetical; it was not worth thinking of. The earnest arguments, I admit, favored the permanence of the succession of forms” (translated from de Candolle, 1882).

These thoughts from de Candolle further prove that, with Darwin’s *On the Origin of Species*, the idea of evolution became increasingly more favored than especial creation. The explanatory capability of natural selection was so broad that it won over more and more naturalists. Through this transition, the geographic distribution of organisms became a key factor (Bowler, 1989).

The historical analysis of biogeographic ideas is brought back in the 20th century, when biogeography reaches its acknowledgement as a self-sufficient discipline and reaches the status of an analytical science, with its own conceptual and methodological corpus, which Ball (1976) calls the analytical period. The sequence according to which we present the historiographical analyses on biogeography follows a chronological order.

### Extensionism versus permanentism

Martin Fichman’s work (1977) is a microhistory of the debate in the mid-19th century between extensionism and permanentism. This debate confronted those naturalists that appealed to the past existence of ancient earthly extensions to explain the geographic distribution of organisms (extensionism), particularly the disjunct distributions, and those who argued that the oceanic and continental configuration had not changed in a noticeable manner (permanentism). Extensionism as an idea gained further popularity following the works of Edward Forbes (1846), who proposed the existence of several ancient connections to explain the biotic similarities between England and other areas. Fichman (1977) focuses his attention mainly on the ideas of British naturalist Alfred Russel Wallace (1823–1913) regarding this controversy.

Fichman (1977) analyses the precise shift from extensionism to permanentism in Wallace’s work. In his early works, Wallace explained the biotic similarities amongst areas via hypothetical terrestrial bridges and stated that the idea of an accidental dispersal had been undervalued. In contrast, in *The Geographical Distribution of Animals*, Wallace (1876) no longer mentioned his extensionist hypotheses. Fichman attributes this shift to Darwin’s influence, who firmly and consistently opposed the bold conjectures of the ancient unions of continents. In this regard, Bueno and Llorente (2004) point out that Wallace’s change of stances occurred in an abrupt manner. On June 1863, his work *On the Physical Geography of the Malay Archipelago* (Wallace, 1863) was read it holding a clear extensionist position in the *Royal Geographical Society.* Just two and a half months later, his work *On the Geographical Distribution of Animal Life* was also read at the meeting of zoology and botany of *The British Association for the Advancement of Science* and was published next year (Wallace, 1864), although this time with a clear rejection of extensionism.

### Neither darwin nor wallace, but buffon and augustin de candolle

Gareth Nelson (1978) conducted his own history of biogeography with the intent of redeeming pre-Darwinian naturalists as pioneer biogeographers. Nelson’s basic argument is that there are general principles and concepts that mold the identity of this discipline, originated mainly in the work of Augustin P. de Candolle in the second decade of the 19th century. This way, he also criticizes the leaning history brought forward by dispersalist biogeographers, namely Matthew, Darlington, Simpson and Mayr, which he considers a Whiggish history that glorifies Darwin and Wallace as the fathers of biogeography. Even back in the decade of 1830, both James Cowles Prichard (1786–1848) and Charles Lyell (1797–1875), acknowledged that Augustin de Candolle had clearly proved that the physical conditions of the environment did not define species distribution. Nelson tracks the origin of that idea down, which involves the rejection of the old ecological determinism of the design doctrine (Nelson, 1978). He maintains that Georges-Louis Leclerc, Comte de Buffon (1707–1788) was the first to formulate it back in 1761 on an empirical bases, and that Augustin de Candolle developed it by making the conceptual distinction between physical causes that affected the spatial distribution of organisms only on a local level (stations), and historical causes, responsible for large distribution patterns (habitations). The environmental conditions were clearly insufficient to explain organism distribution. In Nelson’s reconstruction, Darwin and Wallace are not the founders of biogeography, like the dispersalist hagiography affirmed, but rather Buffon and Augustin de Candolle are granted this role. He admits, however, that this is not an in-depth study, as he did not cover the period between Buffon and de Candolle (Nelson, 1978).

Nelson makes a sharp critique of the research program developed under the dispersalist approach, in which the centre of origin and the dispersal routes of each taxon are searched for, leading to explanations that neglect general biogeographical patterns. He maintains that the modern equivalents of the concepts of station and habitation have become ecological and historical biogeography, respectively. Nelson acknowledges that from the approach of Augustin de Candolle’s work, the central subject is the research of nature and the causes of biogeographic regions. In turn, this approach is a consequence of the generalization de Candolle made regarding Buffon’s law, according to which there are different species in different areas, regardless of environmental conditions. Because of that, Nelson maintains that Buffon’s Law serves as the starting point of the study of the geographical distribution of organisms, and that the research programs that developed in the second half of the twentieth century (panbiogeography and vicariance approaches) ultimately stem from the same law (Nelson, 1978).

### Biogeography and darwinism

The reconstruction Richardson (1981) makes is, like Fichman’s, a microhistory, though Richardson focuses on the biogeographic evidence Darwin used to support his own transmutation theory. Based on the *Collection* of Darwin kept at the library of Cambridge, Richardson reconstructs the development of the biogeographic conception of Darwin, who keenly studied the geographic distribution of organisms, motivated by his great interest in researching the creation of races, human in particular. Richardson’s main interest is to prove that biogeography provided Darwin with the most solid empirical evidence in favor of his theory of the descendent with modification and was the basis on which he doubted the fixity of species, their perfect adaptation to the areas in which they lived and the belief of multiple origins of the same species or representative species in different time periods or locations.

Richardson (1981) analyzes the evidence Darwin used in favor of his theory. The first one is shown in *On the Origin of Species* and refers to the physical conditions of the environment not being enough to explain the biogeographical distribution (Darwin, 1859). Darwin was intrigued by the absence of species in areas with adequate habitats for them, particularly islands. While the hypothesis of species transmutation was enough for Darwin as an explanation for these biogeographic events, for some theist naturalists, such as Swiss naturalist Louis Agassiz (1807–1873), these were the empirical evidence in favor of the theory of multiple creations and the direct intervention of God (Agassiz, 1850). This is a case where the empirical data seem to be neutral to different interpretations.

Richardson determines that it was between 1838 and 1839 that Darwin established the connection between his natural selection theory and the geographic distribution facts. Later, in his *Essay* in 1844, he clearly established natural selection as the *vera causa* of species transmutation, and of biogeographic distribution as the main proof of his theory (Richardson, 1981).

Both Michael Paul Kinch (1980) and James Larson (1986) agree in their interest in rescuing and analyzing the ideas that were discussed around the geographical distribution of organisms in the 19th century. Despite the influential idea according to which, before making great theoretical developments, the pertinent thing was to make an exhaustive inventory of the creatures that inhabited the earth, they identify the main questions that scholars of distribution were asking and clearly agree. The main theoretical aspects being discussed were: (1) whether the human species had a monophyletic or polyphyletic origin, (2) how stable species were, (3) what the role of God was in the creation of species, (4) whether there was a design in nature and how it could be unveiled, (5) which was the most adequate concept of “species”, (6) whether similar species inhabited similar climates, (7) was there one or several centres of creation?, (8) was there a system of regions that had universal validity?, and (9) how could disjunct distributions be explained (dispersal, independent creations, climate similarity, or ancient connections)? It is evident that the resolution of these questions involved both empirical work and theorizing.

Kinch’s (1980) objective is to analyze the connection between the studies of the geographic distribution of organisms and these theoretical discussions. He makes a distinction between two opposing points of view to explain the geographic distribution of organisms. At one end of the spectrum, there were those who claimed that biogeographic regions were permanent and had been created by the direct intervention of God, for example, Philip L. Sclater (1858), Louis Agassiz (1850) and William Kirby (1835). Those with more moderate views, such as Karl Willdenow, only implied their approval of design, though its purpose was earthlier: to discover the different creation areas with empirical data. Others, like Prichard and Lyell, explicitly separated themselves from direct metaphysical interventions by leaning towards an explanation model that appealed to natural laws. God had only set the world in motion but was no longer intervening in its functioning (Kinch, 1980).

It is clear that these theoretical discussions were an important influence on the work of scholars studying the geographical distribution of living beings. The case of disjunct distributions was the most controversial for both deist and naturalist stances. If the design of the world involved fixed and immutable species and distribution areas, disjunct distributions implied independent origins of the same species in different areas and it became pointless to attempt to explain their presence by natural causes, either by accidental dispersal or by no longer existing land straits; for this same reason, it was also pointless to look into the origin of species. This debate had reached an impasse that was not broken through until after Darwin’s theory (1859). Kinch analyzes how monogenetic and polygenetic theses on the origin of the human species led the biogeographic discussion. Like this, the rejection of a literal interpretation of *Genesis* by Karl Willdenow, James Cowles Prichard or Philip Lutley Sclater ultimately replaced the traditional belief in a single centre of origin for a newer one that accepted different centres of creation. The utilitarian vision of natural theology had an influence on naturalists like Kirby to develop explanations about organic distribution. On top of this, and alongside natural theology in its formal version like Agassiz, it encouraged naturalists such as Alexander von Humboldt and William Swainson to look for principles of the natural order of the creation.

Kinch (1980) argues that the dynamic vision regarding Earth, supported by Lyell, changed the conception of biogeographic regions that before his time had been perceived as essentially static entities that represented different areas of creation. Lyell acquired an interest in organic distribution and its possible explanations that could be given in accordance with uniformitarian principles in geology. Kinch points out that an important debate that took place in the mid-19th century, was if biogeographical regions were creations that had been directly designed and executed by a divine intervention, or if they could be explained as result of natural phenomena. This explanation conceived biogeographical regions as changing entities, the result of frequent dispersal events that depended on constant geological changes. The physical barriers that allowed or impeded dispersal kept appearing and disappearing. Thus, biogeographical regions only had a relative state of permanence. The main concern of this debate was to find an explanation for disjunct distributions.

Janet Browne’s work (Browne, 1983) can be considered as a deconstruction of the biblical myth of the dispersal of organisms from Mount Ararat, which constituted the canonical explanation of the geographic distribution of organisms. Browne leans towards a history of ideas, even if it tackles aspects of the praxis of experts in the field of the geographic distribution of organisms, redeeming several pre-Darwinian naturalists and highlighting their contributions to understanding geographical distribution. Browne alludes to the Jesuit Joseph d’Acosta (1540–1600), one of the first enlightened to visit the New World, and since the 16th century, questioned the biblical explanation of the geographical distribution of organisms. He first addressed the issue on what caused the presence of peculiar species in the New World that could not be found in the Old World, doubting their possible dispersal originating from Mount Ararat, in the extreme east of Turkey. Browne looks into the works which dismissed the idea of organism dispersal from one single centre of origin, such as those from Johann Reinhold Forster, Eberhard Zimmerman and Karl Wildenow, who leaned towards the existence of several centres of origin. Zimmermann even reasoned that the idea of species inhabiting the same area from which they originated was more logical than their dispersal from a different location. Similar to Nelson, Browne acknowledges Buffon’s relevance, who noticed that different species inhabited different areas. Browne highlights the pervasive effect that the work of Humboldt had, who with an integrative approach, tried to bring together politics, economics, and science, along with his input of botanical arithmetic to study the geographical distribution of plants. Browne notes that the mathematization of science was a powerful trend in the first half of the 19th century. Darwin himself, who was an admirer of Humboldt and familiar with his botanical arithmetic, did not shy away from this trend. Browne also states that botanical arithmetic was the context from which Darwin derived the principle of divergence (Browne, 1980). When facing the Whigghish thesis developed by neo-Darwinian biogeographers, such as Philip J. Darlington, Jr. (1904–1983), who claim that scientific biogeography emerged with Darwin and Wallace, Browne highlights the work of a series of previous naturalists, making it clear that both Darwin and Wallace were able to surpass the ecological determinism of natural theology thanks to the achievements of these naturalists. In this regard, Browne agrees with Nelson, who had made these remarks previously. Browne concludes that Darwin and Wallace were not the first authors to question the biblical explanation of the geographical organism distribution; actually, there were several naturalists who discredited Noah’s Ark. A distinctive feature of Browne’s work is that she remarks mainly on British authors as protagonists of the history of biogeography and omits others from other countries.

In his influential work on the development of the evolutionary paradigm, Mayr (1982) lays out an intriguing summary of the history of biogeography. The subtitle itself (Common descent and patterns of geographical distribution, p. 439) states its central thesis right away: biogeography only reached its peak with the biogeographic ideas laid out by Darwin. Like Richardson (1981), he recognizes that the patterns of organic distribution served as the most substantial empirical support for Darwin to develop his theory on descendent with modification. According to Mayr, biogeography became relevant only by its aid for the development of Darwin’s theory. He marks Buffon’s contributions as noteworthy, who, in his attempt to separate himself from Linnaeus’ ecological determinism, clearly stated that the physical conditions were insubstantial evidence to explain biogeographical patterns. He also credits Alphonse de Candolle as the main scholar on the topic of disjunct distributions and notes that, despite his initial creationist stance, he ultimately leaned into historical causes for their explanation, specifically, the occasional dispersal of the past.

After the publication of *On the Origin of Species*, de Candolle himself admitted that “the theory of a succession of forms by deviations of anterior forms” was “the most natural hypothesis” to explain disjunctions (de Candolle, 1862, cited by Mayr 1982, p. 445). Mayr, like Richardson and Kinch, recognize that disjunct patterns caused the most debate among biogeographers. That is why he turns his attention to the work of the Forsters, who explained this pattern by climatic similarities, to contrast it with Darwin’s explanation, who attributed its cause to extraordinary dispersals. Mayr refutes the theistic fundamentalism of Louis Agassiz and compares it with the vision of Darwin, whose fundamental contribution was to have finally taken the decisive step to free biogeography from creationist assumptions, with a simple idea but with great explanatory power: geographical distribution of organisms was a consequence of descent with modification. In his eagerness to glorify Darwin, however, Mayr overlooks the fact that half a century earlier, Augustin de Candolle had already studied biogeography from a clearly secular perspective, even when the Darwinian theory did not exist. Mayr highlights the methodological rigour from chapters XI and XII of *On the Origin of Species*, and considers Darwin as the founder of casual biogeography, as he was more interested in the study of the causes of distribution rather than in its patterns.

An outstanding element in Mayr’s history is his eagerness to prove Darwin’s stance regarding the stability of the Earth’s surface. A follower of Lyell’s actualist tradition, Darwin interpreted distribution according to the current configuration of the continents and opposed the reconstruction of land bridges, contrary to Forbes and most biogeographers for the next 80 years. Darwin’s explanatory model is based on two premises. The first is that each species was introduced to a single region; the second is his rejection of the climatic determinism of the design doctrine. Matthew (1915), Simpson (1940), Mayr himself (1944), Darlington (1957), and Carlquist (1974), among others, maintained Darwin’s permanentist position (*sensu* Fichman 1977), crediting the dispersal and rejecting hypothetical bridges. This position can be understood in writings prior to the general acceptance of the theory of continental drift, although it is more difficult to understand Mayr’s persistent disregard of this theory and its consequences for explaining biogeographical distribution.

Although Mayr acknowledges that Wegener’s theory of continental drift gained traction after the 1960s, when plate tectonics theory emerged, he is not very enthusiastic about it. He admits that it could explain patterns originating in the Cretaceous and Jurassic, such as the distribution of the main groups of freshwater fish (in a veiled reference to Gareth Nelson, a paleoichthyologist); however, it could not explain other patterns, such as the greater affinity of Australian birds with those of Asia and not with those of South America, despite the fact that the continental drift model assumed that Australia had separated from South America at the beginning of the Tertiary. Mayr concludes that the history of the Pacific remains controversial and is not clearly explained by the continental drift theory.

According to Mayr’s reconstruction of the history of biogeography, the theory of descent with modification plus Darwin’s two explanations for disjunct distributions, i.e., long-distance dispersal events and the extinction of intermediate populations, they are sufficient to explain any case of distribution. He thus justifies that the protocol of biogeographic research has been since then the study of the barriers and the dispersal capacities of plants and animals. In this way, Mayr endorses the central role of dispersal that Darwin assigned as the main cause of the geographical distribution of organisms and ignores any reference to vicariance. In a clear self-reference, Mayr affirms that Darwin was so advanced in his biogeographical conceptions that only until the 1940s could they be assimilated.

Darwin was not concerned with the approach put forward by Sclater (1858), who suggested the research on which were the main biogeographic regions and their relationships with each other, which Mayr dismissed as static and descriptive. Sclater’s approach, however, prevailed through the following decades, led by Alfred R. Wallace (1876). The regionalization studies were carried on at a finer scale with Alphonse de Candolle and stretch up to this day. In Mayr’s view, such studies do not cross the threshold beyond a descriptive level and have little input in making generalizations.

Peter J. Bowler (1996) made a historical reconstruction of biogeography, which stands out due to the analysis it makes regarding the deep influence that the discovery of fossil remains had on zoogeographic awareness and their evolutionary meaning. It addresses the connection between morphology, paleontology and biogeography, an area of study that had not been as researched back then. In the eighth chapter (*The Geography of Life*) of *Life’s Splendid Drama* (Bowler, 1996), he highlights the role that the study of fossils had on shifting the direction of ecological explanations, which had been relating the distribution of organisms solely to environmental conditions. Paleontology could reveal changes in the geographical distribution of species not only in a spatial dimension but in a temporal one as well. This was particularly promising, as it could potentially reveal the areas of origin for plant and animal taxa, as well as the displacements they had experienced before they reached their current distribution. But above all, the study of fossils and their geographical distribution opened another possibility that was not in the perspective of population genetics or adaptation studies: the reconstruction of the history of life.

Bowler’s research also revolves around the development of evolutionary theory. It tackles the debate between extensionist naturalists, such as Edward Forbes (1815–1854) and Joseph D. Hooker (1817–1911), among others, and the permanentists, like Darwin himself, who rejected the idea of the sinking of huge continental masses having occurred within the recent geological past. Bowler highlighted how the Malthusian principle was widely accepted as a cause of the natural tendency of species towards expansion, even by those opposed to the Darwinian theory of natural selection. He explains how this theory heavily influenced the approval of the dispersalist biogeographic approach, finally replacing its rival concept of *inertia*, proposed by Andrew Murray (1812–1878). According to Murray, biogeographical regions remained relatively stable because organisms instinctively stayed in their area of habitat, as long as it proved convenient for their lifestyle. Thus, the concept of inertia denied the idea that organisms had a natural tendency towards expansion. In the end, the idea of dispersal had such degree of approval that both extensionists and permanentists ended up accepting it as the cause of the current distribution. Bowler concludes that the debate between extensionism and permanentism reached a dead end, as the advocates of each posture could accuse the opposite side of falling into ad hoc speculations. Permanentist theorists could always aid themselves with the hypothesis of improbable dispersals, whereas extensionists could always hypothesize the existence of some sort of terrestrial bridge for any case of disjunct distribution.

Bowler also approaches the debate regarding the idea of an independent evolution, also known as parallel evolution, which contradicted the Darwinian biogeographical model. Two of the central points of Darwinism were that (1) each species had originated just once, and (2) that the spatial disposition between continents and oceans had remained unaltered, at least since the Cenozoic Era. The spatial consequence of these principles was that the only way to explain the current geographical distribution of species was their dispersal from their centre of origin. Naturalists such as Angelo Heilprin (1853–1907) and Richard Lydekker (1849–1915), amongst others, proposed the idea that the same species could have originated independently in different areas. This way, the concept of parallel evolutions simply deemed Darwin’s model pointless. The search for centres of origin and dispersal routes would be of no use.

Bowler concluded that the conjunction between evolutionary ideas and research on current and past organism distributions towards the end of the 19th century and the early 20th century was a decisive influence on the predominant research in biogeography. It consisted in finding the location where groups of organisms had evolved and accepting that any successful species would have the automatic tendency to expand its territory as much as possible. Thus, the Holarctic distribution model was structured, according to which the more evolutionary advanced groups had originated in the most hostile northern climates. The periods of climatic stress had triggered successive waves of invasion of the superior northern groups that would later extend towards the south, displacing or wiping out the ancient groups (Morrone, 2022).

### History of practices in biogeography

James Larson (1986) analyzed the research performed in the late 18th century on “geographical history”, namely, the spatial organization of the organic beings. Nelson (1978) noted that he had not addressed the time frame between Buffon and de Candolle, which could be an interesting venture. Larson’s work is an important contribution spanning across this period of time, one where he analyses the works of North European naturalists which had been neglected up until then. Larson realized that the problems of the spatial distribution of life were secondary in respect to the main interest, which was to make the inventory of the living beings and to build a natural system to properly organize them.

in practice, they subordinated organic distribution to purely formal relations, the affinities and analogies of systematics. In neither case did geographical history emerge as an independent form of discourse. (Larson 1986, p. 447)

It is worth noting that Larson clearly differs from Nelson on this regard, who argues that zoogeographic issues took a central role within the anti-Linnaean and anti-essentialist conceptual frame that Buffon developed. Larson believes that Nelson’s proclamation of Buffon as the father of biogeography to be excessive, as the latter limited himself to the detailed observation of the wildlife differences between the New and Old World, comparing only mammal species, without properly elaborating on a more polished biogeographic account (Larson, 1986).

According to Larson, Nelson’s rendition, which marks Buffon’s law as the original cause from which the panbiogeography research programs emerge, and the vicariance model are both taken out of context. Larson dismisses this statement as anachronistic and presentist. Nonetheless, Larson identifies two theoretical perspectives for the study of the geographic distribution of organisms: an essentialist one, originating in the works of Linnaeus, and a more traditional and eclectic one, developed by Buffon.

Historical geography, as understood by naturalists in the late 18th century, was the methodical study of the factors that affected the geographical distribution of animals and plants across the globe, as well as the general principles that ruled them, which clearly makes this discipline the predecessor of biogeography. Larson, however, points out that the context in which the discussion developed was quite different to that of modern biology, which definitely influenced the perception of these factors, their conceptualization and the problems they posed.

Larson acknowledges that the input Buffon provided was relevant and innovative, as it represented an attempt to explain organic distribution through historical processes. Larson, however, points out that the fact that historians have particularly focused on theoretical aspects of Linnaeus and Buffon has resulted in the neglect of the great amount of distribution factors, such as distributional data that were collected and organized in the second half of the 18th century. Therefore, it would be important to also address the historical study of which were the practices of 18th century naturalists. Larson highlights the valuable influence of Eberhard August Wilhelm von Zimmermann’s vision, who carried out the first great work about historical geography, and who maintained an ambivalent attitude towards Linnaeus and Buffon. His work represented a reaction against both Linnaeus’ speculations of both Linnaeus and Buffon. Linnaeus explained the geographical distribution of organisms from a hypothetical original island-mountain, which was the only emerged land. From there, the different species migrated as more land emerged and dispersed until they reached their current distribution. For his part, Buffon explained the distribution of animals from his theory of progressive cooling of the Earth. The first animals arose in the polar regions, which were very hot at the time. As the Earth cooled, animals spread progressively to temperate and equatorial regions. Zimmermann set himself a more modest goal, though it proved to be more accessible: to collect empirical facts, given that before assembling any theory, more and better information was required on the issues of distribution and physical conditions on a global scale. With the imperfect information that was available, it was only possible to speculate on matters such as potential dispersal events, adaptability to new habitats, or past geographical conditions. Several naturalists were influenced by the approach Zimmerman took, and consciously attempted to avoid any speculation. It was only towards the late 19th century that the interest in theorization reemerged, though more information was provided at this point.

In an outstanding and relatively recent work, Malte Ebach (2015) develops an original and interesting version of the history of biogeography, which matches up with Larson’s proposal to develop a history that emphasized on the study of the practices of biogeographers, rather than on their ideas. Ebach makes a critical analysis of the histories of biogeography, declaring that many of them were mainly conceived based on the beliefs of their practitioners, especially those related to the development of evolutionary theory. Ebach stresses the fact that history has rarely been told based on an approach to the actual work of biogeographers. His intension, therefore, was to incorporate a history of the practices and methods used by phytogeographers and zoogeographers from 1770 to 1890 s (Ebach, 2015, p. vii), without drawing upon the histories made by historians, but choosing to appeal to primary sources from the naturalists of these centuries instead. This way, he presents his work as an alternative history, mainly directed towards practicing biogeographers. However, the exclusion of the interpretations of historians and his emphasis on primary sources do not exempt his interpretation from being just one more in the history of biogeography.

According to Ebach, what determined the means to perform biogeographic regionalization was the type of classification practice. In the 19th century, two important schools had developed, one of Linnaean lineage and the other with a Humboldtian approach. The first one sought for taxonomic criteria while the second one focused more on physiognomic criteria. Ebach argues that the practice and specialization of classification were what guided the course biogeography took in the 18th and 19th century. This goes against a popular idea among biogeography historians, according to whom the development of evolutionary concepts was essential to the design of this discipline. He denies that evolutionary theory was the outcome of the plant and animal geography from the first half of the 19th century. Naturalists were stuck trying to establish a way of classifying organisms rather than looking for a theoretical synthesis of the factors of the organic distribution.

Ebach scorns the glamorous histories of biogeography, with founding fathers and clear origins of the discipline, which, by the way, do not have a “single centre of origin”, which is why the attempts to unify it have always been to no avail. He also criticizes the histories that establish illustrious founders of the discipline, such as Humboldt, Wallace or Darwin, as their glorification implies hidden fallacies, as well as anachronisms of terms and concepts associated with these so-called founding fathers. The term “biogeography” itself was not coined until the late 19th century, and by extension, it appeared later than the works of these naturalists. It is assumed that the intellectual lineage of characters such as the previously mentioned experts lingers even nowadays, which appears to give a Whiggish tinge to these histories. Because of this, an anachronic identity as a proper and autonomous discipline, though historically fictional, is assigned to biogeography.

Ebach argues that in Nelson’s version of the history of the biogeography Buffon and de Candolle appear as founding fathers because of Nelson’s exclusive interest in historical biogeography. According to Ebach, Nelson carried out, as referred to by Larson, a “selective reading” on Buffon, which resulted in his own history of the biogeography to become slanted. The contribution of Buffon to biogeography was not so relevant, as he used geographic distribution to explain disjunct distributions, which makes it, in the eyes of Ebach, a law that is more based on taxonomy than biogeography. To complete his criticism of Nelson´s work, Ebach points out that Buffon’s input was not even original, as the concept described as “Buffon’s Law” had been previously addressed by Linnaeus.

## Discussion

The reviewed works provide different interpretations on the history of biogeography. Two initial approaches stand out. The first, led by Mayr (1982), comes down to the continuation of the history of biogeography put together by the dispersalist biogeographers. In his abridged reenactment, Mayr affirms that Darwin’s theory, including his explanation on disjunct distributions (via long distance dispersal events and the extinction of intermediate populations), is enough to explain any distribution instance, though it would not be taking the resolution of minor details into account. The second, Richardson’s work, can be included within the same vein, both because his main concern is the reconstruction of Darwin’s use of biogeographic data as more solid empirical knowledge to support his theory, as well as the compatibility of his views with Mayr’s. Richardson (1968) had already stated that Darwin’s big picture is aimed to figure out the geographic distribution of species, besides some revision to the details still remained valid (Nelson, 1978). The opinions of Mayr and Richardson are essentially the same that Wallace (1876), held from the second half of the 19th century when he noted that the dispersal model of the dominant northern faunas already served to make sense of the basic biogeographic patterns, leaving only the occasional anomalies to be explained.

The interpretations of Mayr and Richardson clearly clash with the reconstructions put forward by Nelson and Browne, who agree at least on one aspect, that being the research into the biogeographic work performed by pre-Darwinian naturalists. This shared interest gave way to an interesting, highly nuanced history regarding the intent and motivations that swayed the direction of the works of those naturalists who studied the geographic distribution of the organisms, even prior to the birth of Darwin, and who had not only put forward some of the core questions on this matter, but also had thorough debates about them. The main differences are that Browne’s history reaches its peak with Darwin, unlike Nelson’s. In any case, both make one fact clear: modern biogeography did not start with Darwin. Ebach himself, despite his broad and scholarly research, acknowledges that his history is merely a sketch, or in his own words, a collection of vignettes over a broad and interesting field that meaning the studies on the spatial distribution of organisms, made in the pre-Darwinian past.

Evidently at odds with Nelson’s reconstruction, Mayr undermines the tradition that, since the 18th century, become more interested on the acknowledgement and study of biogeographic patterns than by the processes that they resulted from. Mayr highlights the contribution of the Modern Synthesis biogeographers, such as G. G. Simpson and himself, whom he places within, as he calls it, a dynamic approach which was pioneered by E. R. Dunn (1922) as the means to rebel against the static approach by proposing a casual analysis of the faunas. Mayr portrays Simpson (1940, 1943, 1947) as the head of this innovative perspective within the study of the geographic distribution of mammals and placed himself as the equivalent role for the case of birds. He remarks on the statistical treatment Simpson gave to his studies, which allowed him to gauge dispersal probabilities. In his distinctive direct style, Mayr was not concerned with the branding of his work as a Whiggish history when he states that Darwin was ahead of his time, proven by the fact that his biogeographic ideas were not properly revisited until the 1940s, in an overt instance of self-reference. By this logic, Mayr’s undervaluing Wallace’s work becomes more understandable, dismissing him as merely descriptive. He notes that the key aspect in biogeography research has long been the study of dispersal. In this regard, an additional contrasting note emerges between Browne, who portrays a Darwin with a definite interest in the study of biogeographical patterns, as demonstrated in his works on botanical arithmetic (for a more detailed account of this idea, see Browne 1980), and a disdainful Darwin, as portrayed by Mayr. Taking Mayr’s disdain for pattern studies into account, it comes as a surprise that it was Philip L. Sclater, with his static approach and overt theist conception, who put forward an early Popperian proposal of putting the universal validity of the biogeographic regions to the test.

Sloan (1985) made a thorough analysis of Mayr’s (1982) book, which can be applied to the latter’s section on the history of biogeography. He observes that Mayr has never embraced a historical approach nor has he claimed to understand the past in his own terms; he does not intend to provide a “rational history” sensu Lakatos or a history which agreed with the normative methodological aspects of history. Instead, his intent is to analyze and clarify certain concepts, as an attempt to unravel the string of complex concepts; his motives are not as driven by history as they are by conceptual clarification. Mayr’s history is more normative than descriptive, aiming to select the problems and individuals that gave way to the clarification or haziness of these concepts first. Sloan merely analyzed his own work through the premises of Mayr himself and thus managed to bring forward a convincing narrative that was aligned with the direction of his analysis. Following in the footsteps of Popper, he adheres to the “strong assertion and refutation” technique. Even though Sloan (1985) finds the historical incursion of Mayr refreshing within the thick domain of philosophical-methodological discussions of historians, he ultimately concludes than, in spite of Mayr’s denial, his reconstruction turns out to be discernibly presentist and linear, as it smoothly culminates in the evolutionary model of the Modern Synthesis.

Ebach gives a *tu quoque* reply to the criticism Nelson directs to the history of biogeography put together by dispersalist biogeographers. It should be noted that in spite of the importance of Nelson’s reconstruction, a sense of continuity and anachronism can be observed, by declaring that the Law of Buffon is actually equivalent with allopatric speciation, a concept that implies a theoretical context that has nothing to do with the ones of Buffon or de Candolle. Ebach (2015) pointing out that the Nelson’s narrative is a Whiggish ad hoc history for his vicariance model, according to which the pioneers of the study of the geographic distribution of organized beings, mainly Buffon and Augustin de Candolle, led with good reason to the rise of the vicariance and panbiogeographical schools, while the dispersal approach remains a mere moment in time.

One more point of contrast emerges by comparing the questions presented by Kinch, Fichman, and Richardson, who all became interested in the ideas that prevailed amongst the 19th century scholars of the living organisms’ geography, with those presented by Ebach, who was disdainful towards them and focused on their practices instead. Either way, the histories analyzed within this work, with Mayr’s being the only plausible exception, represent an important shift. Their reclaiming of the contributions of the pre-Darwinian naturalists that analyzed the geographic distribution of the organisms, mainly with the work of Augustin de Candolle, represents a fundamental change regarding the Darwinian “history-hagiography” formed by dispersalist biogeographers (Darlington, Mayr, etc.) and clarify that modern biogeography did not start with Darwin. Only two of the nine works analyzed are more inclined to the practices of naturalists who studied the geographical distribution of organisms. Both Larson and Ebach have been interested in this latter approach, while others have focused on the theoretical discussion of ideas.

The history of biogeography as proposed by Ebach intentionally focused on practices related to this discipline. His argument is that naturalists back in the early 19th century, were committed to classification work of a descriptive character and, even though they were able to sketch out some causes for the patterns of the geographic distribution of the organisms, they did so with a merely speculative tone without digging any further into them. Certainly, Augustin de Candolle was interested from a young age in the conclusions drawn by Adolphe Brogniart, who realized that plant fossil forms were different to the current ones. Even though these results were not enough for Brogniart to doubt his clearly fixist and catastrophe-leaning stance, Augustin de Candolle was faced with, as one could guess, the canonical ideas arguing that all species had originated in a single centre and had remained static since their creation. However, he refrained from discussing this matter, appealing to the uncertainty regarding both past conditions on Earth and transmutationist ideas.

Ebach revisits Larson’s highlighting of the neglect of historians towards the works of naturalists in Northern Europe; and his work certainly aids in a noticeable manner to fill this gap. He also agrees with the criticism Larson makes on the work of Browne, in the sense that the narrative of this author is ultimately selective, as Browne focuses on the 19th century while oversimplifying the problems regarding the distribution of the organisms that faced the naturalists of the 18th century. A noticeably biased historiography studying the practices of the experts of geographic distribution, however, would also prove to be faulty. In spite of the declaration of principles of Ebach, the limits he establishes between the concepts and the practices of naturalists appear to be blurred at times. It is relevant to make some remarks on the criticism Ebach makes with regards to histories mainly based on concepts, and particularly on his criticism on the work of Janet Browne, in the sense that she does not tackle the practices of biogeographers and instead narrates the history of biogeography as if it was the predecessor of the development of the Darwinian theory. It seems undeniable that ideas have played a crucial role in the biogeographic conceptions of the naturalists of this time period regarding the geographic distribution of the organisms. For instance, how could concepts such as “flora”, “fauna”, “station” or “habitation” be explained without a theoretical framework? Beginning with the idea that species had originated in the place they inhabit, in contrast with the previous dominant idea that they had dispersed from a single origin centre, a new idea emerges arguing that not only particular species, but entire species groups that dwell in a region make up a larger body of components that are not only spatially coincident, but also form integrated sets of species, that is to say, floras and faunas.

On the other hand, how would it be possible to understand the concept of “habitation” without its theoretical burden? This concept is only possible by rejecting the previous ecological determinism of natural theology. It is difficult to conceive an advance in biogeographical knowledge without theorizing. Or at the very least, without taking the influence of the dominant epistemic framework into account. It was only after the term flora was presented as integrated groups of species that it became possible for a series of works on the flora of several regions to proliferate (i.e., the Flora Lapponica of Linnaeus, the Flora Sibirica and the Flora Orientalis of J. G. Gmelin, the Flore Française of Lamarck, among others). While taxonomic practice was indeed the one that allowed the development of biogeography, as Ebach argues, what incited this same development was the interplay between praxis and theory.

It was only beginning with the theorization that flora and fauna were conceived as living entities that were typical and exclusive of a geographical region. These theoretical concepts captivated the imagination of countless naturalists. Browne convincingly argues that starting with the coining of the ideas of flora and fauna the use of the concepts of biological provinces as virtual synonyms arose. It is only this way that biological provinces are interpreted as integrated communities of organisms, not only bound by their obvious geographical relation, but also by the close bonds among species. In other words, the concepts of flora and fauna transcend the geographical connection between their component species towards a plane that is properly ecological and even historical.

**Fig. 1:**
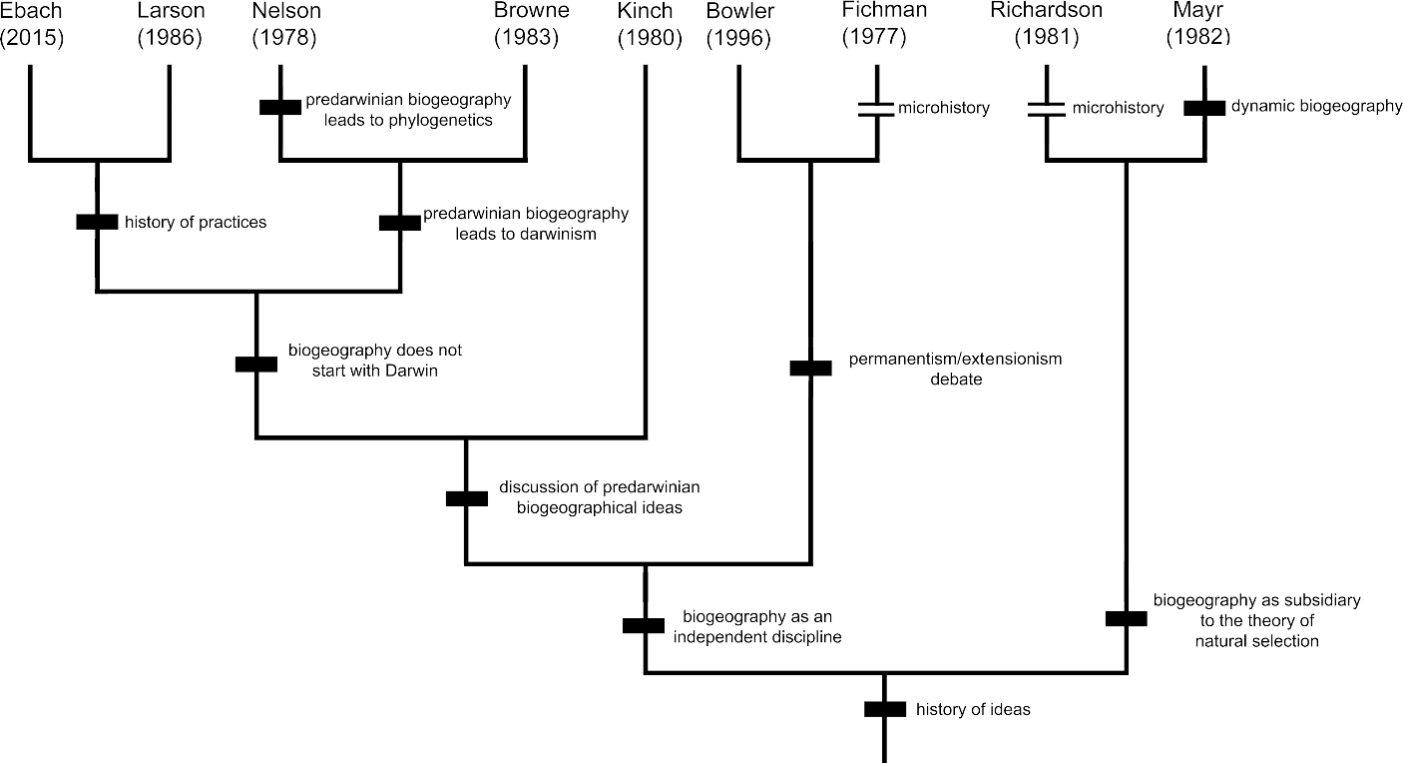
Shared conceptions among historiographers of biogeography depicted as a phylogenetic tree

Although Ebach convincingly argues that the central interest of the naturalists was a taxonomic one, while Larson establishes that the spatial relations between the groups were subordinated to the merely formal relations of the taxonomy, it seems that this did not prevent the collateral development of theoretical representations on the spatial distribution of organisms. We propose a provisional “phylogeny” among the different authors who have been reviewed in this work, in order to summarize their ideas (Fig. 1).

With the concept of biological provinces, a series of investigations that have been ongoing since the 20th century acquire a new meaning, such as searching for their limits, the species that shape them, or their physical and topographic attributes. This is a clear example that opposes the affirmation of Ebach, which argued that the development of biogeography had little to do with theorizations, and that the essential agent for the development of this discipline had been the practices of biogeographers themselves. The very dichotomy that Ebach identifies, between the taxon regionalization of Augustin de Candolle or the physiognomic one by Humboldt, originates from two clearly distinct theoretical conceptions.

Another aspect that arises with the revision of these histories of biogeography is the continuation of controversies on the matter from the 19th century to this day. Some debates, such as the fixed species and the creation areas, have been definitely abandoned. Many others, however, remain valid. For instance, Fichman (1977) affirmed that, since the mid-19th century, Wallace denied having given much importance to the dispersal factor in his early works, even though he ended up portraying it as the main cause of the biogeographical patterns. Currently, the relative importance of the processes which gave way to the biogeographical patterns, both current and past, is still under discussion. This dispute itself implies its importance in the current biogeographical research for the study of both the processes and the patterns. Nowadays, the disdain Mayr held for the study of patterns would seem excessive. The undeniable heuristic value that the recognition of patterns holds as a first approach to explain their causes is properly acknowledged.

In the 19th century, August de Candolle looked for “regions” and Humboldt for “assemblages”, and the interest to research zoogeographic regions, areas of endemism, geographic pattens in species richness, and phylogeographic structures remains in the 21st century. The interest in developing a strong theory on biodiversity also implies debates regarding the patterns of practical interest for the preservation of biodiversity, such as the Single Large or Several Small reserves (SLOSS) debate, habitat corridors, metapopulation theory, and nestedness (Whittaker et al., 2005).

The balance between the research on processes and patterns leans towards one side or the other, depending on the different case studies involved. Some favor the processes, both current and past, which have generated biogeographical patterns, with dispersal factored in, though they do acknowledge that phylogeography, which ultimately studies patterns of the spatial distribution of genes, has the potential to differentiate between the relative worth of the vicariance and dispersal models in the composition of the current biogeographical patterns (McDowell 2004, 2008).

## Conclusions

A common aspiration amongst scholars of the spatial distribution of living organisms remains, that has trascended through generations, is the desire to reclaim the history of life on Earth, which has become the root for several controversies that have prevailed since the 19th century and which are still present today, within the biogeographic research as well as in the historical research of biogeography.

History exists in a perpetual state of renovation. The histories of biogeography put forward by the reviewed authors had their own motivations; with Nelson’s having the boom of cladistics as a backdrop; and alongside with Janet Browne, they pinpoint the beginning of the history of the recently recognized biogeography and assert it as a self-sufficient science, not as a mere instrument used by Darwinism, as the neodarwinian hagiographic history claimed. There is no central dogma nor dominant paradigm within the discipline of biogeography, which may explain why several biogeography histories have been developed through time. In addition, we agree with Ebach (2015) that none of the authors of the histories of biogeography, including those reviewed in this work, followed a standard method or engaged with the literature of the history of sciences. It is necessary to recognize the emphasis that Ebach puts on the diverse nature of biogeography, in the sense that it is necessary to assemble a more complete history of biogeographic practices; however, despite the importance of social studies of science, it is difficult to make out how the scientific practices are able to detach themselves from a theoretical framework, no matter how general the latter is.

The internalist histories of biogeography have had the virtue of displaying the importance of this discipline, by bringing forward a constant core question within biology: where and how did species originate? It is this precise link, which explains why several historiographers of biogeography ended up developing their narrations around the arrival of the Darwinian theory, as it was ultimately the one that provided a persuasive answer to the “mystery of mysteries” (Darwin, 1859). Aside from the inherent worth of the study of the history of biogeography, it also serves as a reference to reach a wider and more informed vision regarding the debates that have and still are taking place to explain the spatial-temporal distribution of biodiversity. This work served as a proposal for a vision that provides a more balanced version, where both internalist approach as histories of praxis are presented as valid. Contrary to what Ebach thinks, ideas have always been a relevant factor for the development of biogeography. All the historiographical analyses considered, except Ebach’s, have been made according to ideas, theories; however, we still lack social histories of science, where the influence that experiments, methods, techniques, devices, politics and culture have had on the constitution of biogeography as an autonomous discipline. Ebach’s contribution leaves open interesting perspectives to carry out this type of research.

Like in any phylogeny, the evolutionary history of historiographical approaches can be depicted on multiple dimensions, but only a few can be recovered. We can look into the sociopolitical environment and cultural, social, and academic origins of authors and prevalent points of view in the academic community. How much an author influences others is a matter of hard evidence, possibly from letters or explicit references in the papers. So, ancestry ties in ideas and points of view are hard to set. Practitioners and historiographers complete the scenario where the history of science has run and made its track. No history can be considered complete since some authors are inevitably excluded. In this work, we had to leave aside authors like Nils Gustaf Erland von Hofsten (1874–1956), who presented the first detailed history of the geographical distribution of living beings from Aristotle to Daniele Rosa (Ebach, 2006); Evgenii Wulff (1943), whose primary interest was in explaining the distribution of organisms (Ebach & Humphries, 2003); Erik Nordenskiold, that interpreted Humboldt’s plant geography and equated it to ecology (Ebach, 2015); and Nelson Papavero (Papavero 1997, 2003; Papavero & Llorente-Bousquets 2007), who provided several chronological narratives covering the pre-evolutionary biogeography. Papavero’s work rivals Von Hofsten’s for its breadth and depth (Ebach, 2015). It would be worth delving into the contributions of these authors.
